# Great Balls Against Food Waste—An Innovative Nudging Intervention Method

**DOI:** 10.3390/foods15132291

**Published:** 2026-06-26

**Authors:** Jan den Boer, Milena Cygal, Karolina Sobieraj, Emilia den Boer, Gudrun Obersteiner

**Affiliations:** 1 Department of Applied Bioeconomy, Wrocław University of Environmental and Life Sciences, 50-375 Wrocław, Poland; jan.denboer@upwr.edu.pl (J.d.B.); karolina.sobieraj@upwr.edu.pl (K.S.); 2 Faculty of Environmental Engineering and Geodesy, Wrocław University of Environmental and Life Sciences, 50-375 Wrocław, Poland; 124520@student.upwr.edu.pl; 3 Faculty of Environmental Engineering, Wroclaw University of Technology, 50-370 Wrocław, Poland; emilia.denboer@pwr.edu.pl; 4 Institute of Waste Management and Circularity, University of Natural Resources and Life Sciences, 1190 Vienna, Austria

**Keywords:** food waste, school catering, plate waste, unserved food, school canteens, food waste interventions, nudging strategy

## Abstract

Food waste is one of the most pressing obstacles to sustainable development. Reducing food waste in schools and kindergartens constitutes an important component of sustainable waste management. To achieve this reduction, various interventions targeting food waste can deliver multiple benefits across environmental, social, and economic dimensions. Among these, behavioral “nudges” aim to steer consumer choices without restricting options. This study evaluated a novel nudging intervention in the canteens of two primary schools and one kindergarten, with the goal of reducing plate waste. The nudging intervention consisted of a simple, interactive installation designed to encourage children to reflect on their food consumption and portion choices. The installation was integrated into routine lunch service and it combined ball-based voting with visual prompts: the emptier the returned plate, the greater the voting weight for the pupil. Across all institutions the food waste level (soup and second dish combined) was significantly decreased during the nudging intervention: by 31% for primary school no. 84, 18% for school no. 1, and 33% for kindergarten no. 56, although part of this reduction was attributable to lower food production volumes. Plate waste for the second dish decreased in all the considered schools: by 10 g/meal (11%), 19 g/meal (22%), and 52 g/meal (51%), respectively. After the intervention a larger share of the second dish served was consumed than was left on the plates compared to the situation during the baseline monitoring. A shift from plate waste to unserved food, which was one of the goals of the study, could not be unambiguously confirmed. Overall, the new nudging installation appears effective. Substantial changes in food production complicate the possibility of determining the effects of the nudging intervention. Future research should maintain constant production levels across the baseline and intervention periods. In addition, pupils should be given maximum freedom to determine their portion sizes during the nudging intervention. The long-term effects of the nudging approach should also be evaluated.

## 1. Introduction

Food waste is one of the most pressing challenges to achieving sustainable development in the modern world, as it affects all three dimensions of sustainability and poses increasingly significant ethical, environmental, and economic problems [1,2]. According to estimates, the annual value of wasted food exceeds USD 1 trillion [3]. This corresponds to more than one-third of all food produced for human consumption worldwide [4,5]. Within the European Union alone, over 58 million tonnes of food waste is generated annually—equivalent to approximately 129 kg per capita [6]—with an estimated market value of EUR 132 billion [7]. This problem becomes even more alarming when contrasted with the 783 million individuals affected by hunger and the 150 million children whose cognitive and physical development is hindered by chronic malnutrition, conditions that further deepen social inequality [8,9].

Food waste occurs predominantly in the consumption phases of the supply chain [10]. Households are responsible for more than half of the total food waste in the European Union (53%), with a further 11% generated in restaurants and other food services. The remaining 36% comes from earlier stages of the food supply chain: 19% originates from food and beverage production, 8% from retail and other distribution, and 10% from primary production [6].

Among these sources, school canteens and kindergartens warrant particular attention. Although they are still relatively little researched, they contribute to the overall food waste generation [11]. According to the literature, in the United States, approximately 45% of the food served in elementary schools is wasted [12]. In European primary schools, the average level of avoidable plate waste has been estimated at around 107 g per student per day [13], while in the United Kingdom, the value of food wasted in schools accounts for approximately 26% of the total food budget [14]. More recent studies conducted in preschools in Colombia involving 165 children aged between 6 months and 3 years revealed that the highest levels of food waste occurred in fruits and vegetables, with 17% remaining uneaten, followed by protein-rich foods (15%) and cereals and tubers (10%) [8]. Similar patterns were observed in a study conducted across four kindergartens in Chile, where vegetable losses exceeded 20% in 31% of the institutions, while fruit waste was even higher, with pears identified as the most frequently discarded fruit, recorded in 75% of the study centers [5]. Similar concerns have also been reported in the Republic of Moldova, where, despite strict national catering standards for preschool institutions, recent assessments estimate that nearly 180,000 tonnes of food are wasted annually [2]. In Italy a recent study in 78 primary schools showed that 22% of the prepared food is not eaten, with the total amount of wasted food amounting to 117 g per pupil per day [15]. Likewise, a study conducted in 40 public and private kindergartens in Russia found that early childhood education institutions discarded between 1 and 40 tonnes of food per year [16]. Taken together, these findings highlight the scale of food waste in preschool settings and emphasize the importance of addressing this issue.

Reducing food waste in schools and kindergartens constitutes an important component of sustainable waste management strategies. Interventions targeting food waste in educational settings can deliver multiple benefits across environmental, social, and economic dimensions. These include improvements in students’ dietary intake and the promotion of more sustainable consumption behaviors among younger populations [17]. Furthermore, food waste reduction contributes to more efficient use of resources such as water, energy, and land, as well as addressing ethical concerns associated with food losses in the context of persistent global undernourishment [11]. From an economic perspective, decreasing food waste in schools may also result in cost savings for both institutions and households by reducing food procurement and disposal-related expenditures [18].

However, shifting consumer choices towards more environmentally and socially beneficial patterns remains a complex challenge [19]. Addressing this issue requires the coordinated involvement of multiple stakeholders, including public policy institutions, the food service sector, civil society organizations, and academia [20]. At the same time, schools play a crucial role in shaping the attitude and behavior of future consumers. Not only do children’s nutrition-related choices directly influence food waste in school canteens and kindergartens but they are also likely to influence long-term consumption patterns and sustainability-oriented behavior [21].

Recent studies suggest that the food service sector has considerable potential to more actively shape consumer choice architecture. This can be achieved through the application of behavioral interventions, commonly referred to as “nudges”. This concept, introduced by Thaler and Sunstein in 2009, refers to efforts aimed at influencing individuals’ behavior by engaging their automatic and intuitive cognitive processes rather than restricting or eliminating available choice options [22,23]. Designed to promote beneficial outcomes for both individuals and society, nudges have gradually evolved from a theoretical framework into a widely implemented approach across both public and private sectors [23].

In particular, governments in countries such as the United States, the United Kingdom, and Germany have established dedicated behavioral insight units, contributing to the growing role of nudging in public policy, shaping decision-making processes and influencing everyday behaviors at the population level [24].

Although research in this field has expanded rapidly and empirical evidence points to an increasing use of nudging in various sectors of the global economy, its application in the context of food waste in schools and kindergartens remains limited [25]. Existing studies on nudging have primarily focused on promoting healthier dietary choices [26,27,28], influencing children’s eating behavior in household settings [29], and addressing environmental impacts along the food supply chain [30]. The nudging strategies examined in these contexts typically include the use of labelling and visibility enhancements, sensory cues, and modifications to convenience, portion size, and product placement [31,32]. Regarding food waste in particular, only a limited number of studies have investigated the use of nudging to reduce consumer food waste, with research to date focusing predominantly on household waste [33], as well as institutional settings such as hospitals [34] and hotels [35].

School canteens represent highly controlled environments, offering favorable conditions for the implementation of targeted intervention strategies [21]. Nudging approaches can be integrated into existing educational frameworks to reduce food waste, including initiatives aimed at diverting organic waste-streams from landfills and promoting their conversion into energy and soil amendments through processes such as composting or anaerobic digestion [36]. Given that plate waste has been identified as the primary source of food waste in these environments, nudging interventions at schools and kindergartens should primarily target the reduction of plate waste [17].

In the light of the above, the current study aims to address the existing research gap by examining the application of nudging interventions in school canteens, specifically in primary schools and kindergartens, with the objective of reducing plate waste. The study was conducted across three educational institutions located in Poland, Central Europe, providing empirical evidence from a region that remains underrepresented in the current literature. A novel nudging approach consisting of a plate waste-based voting system was introduced. In the following chapters the nudging system is described in detail, and the achieved results are presented and discussed.

## 2. Materials and Methods

This study was carried out in the frame of the foodCIRCUS project (Circular solutions for keeping food waste out of Central Europe’s schools) within the EU Central Europe program. In the project, pilot actions addressing food waste were structured into four categories: awareness raising, food optimization, nudging strategies, and redistribution. Wrocław University of Environmental and Life Sciences, in cooperation with the Wrocław City Hall, implemented all of these categories in the city of Wrocław, Poland. In the current study, the effects of the nudging intervention are presented.

### 2.1. Aim of the Study

The aim of this study was to evaluate the impact of a novel nudging approach in terms of food waste reduction in educational institutions. Food waste in school canteens is generated both as not served food and plate waste. Whereas not served food (or kitchen surplus) can potentially be redistributed, for example, to charity institutions, the plate waste fraction can only be directed to waste treatment installations. Therefore, specific focus was on the reduction of plate waste.

In terms of the targeted reduction of plate waste, the intervention focused on changing the behavior of the pupils, as they are the ones directly generating this waste (albeit definitely not solely responsible for it). The pupils, in response to the nudge, have two ways to influence the amount of plate waste:

- eat more, thus decreasing the amount left on the plates,
- request smaller portions.

If students request smaller portions, the amount of plate waste will decrease, whilst the amount of unserved food will show an increase. Although the total amount of wasted food would in that case not be reduced directly, it would still be a desirable outcome of the study—the problem of unserved food can be easier dealt with in further measures than the problem of plate waste. A surplus of unserved food can be avoided by producing less or it can be redistributed, for example, to charity organizations.

In the study, both of the above options were examined. In primary schools, some of the pupils do not eat soup but all take the second dish, so, the main focus in the study was on the reduction of the waste arising from the second dish.

### 2.2. Nudging Installation

**Figure 1:**
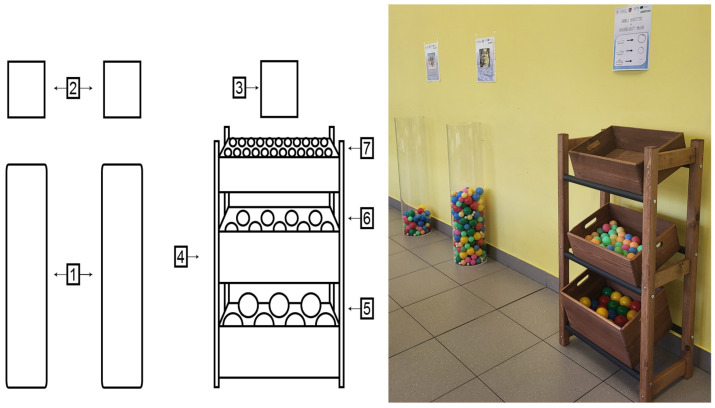
Design (**left**) and realization (**right**) of the nudging installation for primary schools.

The nudging intervention took the form of a simple, interactive installation designed to encourage children to reflect on their food consumption and portion choices. The installation was integrated into routine lunch settings using ball-based voting and visual prompts. The setup consisted of two transparent vertical tubes positioned near the dining area and a stand containing balls of three different sizes. Each ball size corresponded to the amount of food left on a child’s plate after the meal. In Figure 1 the nudging installation is graphically depicted.

For an empty or nearly empty plate pupils received a large ball (5), a medium ball (6) corresponded to a small amount of leftovers, and a small ball (7) was the reward for a nearly untouched plate. After finishing their meal, children were assigned a ball size based on a visual assessment of their plate leftovers and were invited to place the ball into one of the tubes. Thus, the emptier the returned plate, the higher the voting power.

To support correct use, a poster was displayed above the installation, explaining the mapping between approximate plate leftovers and ball size (3). Above the tubes, posters presented the two daily voting options, which changed regularly (2). Examples of applied voting options are as follows:

- Iga Świątek vs. Robert Lewandowski (most famous current Polish female and male athlete)
- I wish I could: fly vs. be invisible
- Pizza or pancakes for the St. Nicholas day lunch
- Two types of candies, to be handed out the next day
- Cat vs. dog (kindergarten)
- Fairy vs. robot (kindergarten)

An additional note placed at the food distribution counter encouraged children to take only as much food as they intended to eat and later vote using the tubes. The intervention did not impose restrictions on food choices or portion sizes but aimed to encourage more conscious decisions through visual feedback and engagement.

### 2.3. Study Design and Educational Institutions Participating

The study was conducted in one kindergarten and two primary schools located in Wrocław, Poland: primary school no. 84 (PS84), primary school no. 1 (PS1), and kindergarten no. 56. (KG56). In each institution, data were collected over five consecutive school days (Monday to Friday), without interruption, in both 2024 (baseline) and 2025 (post-intervention; monitoring was on the same days as the nudging installation was in use in the schools). The research was carried out between November and December.

In the primary schools, meals were served in a common canteen, and the nudging installation was placed in a shared area accessible to all participating classes. In the kindergarten, lunches were consumed in the individual classrooms, where also the installation was located. Kindergarten no. 56 consists of three buildings at two separate locations, with one kitchen functioning in each of the locations. For organizational reasons, it was only possible to monitor food waste generation in one location (kitchen), consisting of two buildings. This implies that to determine the effects of the intervention, the nudging installation was installed in only half of the classes (those classes which had pupils who were present already in November 2024). In total, seven kindergarten groups were included in the intervention. Another seven groups were not involved in the intervention but were part of the food waste monitoring. These classes consisted of different pupils in 2024 and 2025. In 2025 they were new to the location. These classes formed thus a quasi-control group.

The participants were children who regularly attended lunches at the participating institutions. In Table 1 the planned meals and monitoring dates are shown.

No changes were made to standard meal schedules or supervision arrangements, ensuring that observations reflected the typical daily routine. The menus in both monitoring periods were identical. However, pupils were encouraged to ask for portion sizes that they could finish during the intervention period (by a poster at the food distribution window). Kitchen staff were asked to follow the pupil’s portion size requests.

### 2.4. Food Waste Monitoring

Data collection was carried out by members of the UPWr project team, supported by trained student assistants. In primary schools, data were recorded at the institutional level, as meals were consumed in a central canteen across mixed classes and time slots. In contrast, data in kindergartens were collected at the class level, reflecting the decentralized serving system. Both food and leftovers were monitored before entering and after leaving the classrooms.

Food flows were monitored across three stages of the catering process: food preparation (production), unserved food (distinguishing between kitchen surplus and serving leftovers), and plate waste. Other parameters were not measured directly but calculated on the basis of the directly measured parameters. Overall, the following parameters were determined and related to an average meal:

- Planned amount of food (based on the school’s lunch menu, available to parents and pupils, containing both dishes and amounts)
- Production: amount of prepared food—measured
- Not Served: food prepared, but not served—measured
- Plate Waste: food staying behind on the pupil’s plate—measured
- Served: average portion of food served on the pupil’s plate
  Served = Production − Not Served
- Eaten: average amount actually eaten by the pupils
  Eaten = Served − Plate Waste
- Total Wasted: total amount of food not eaten by the pupils
  Total Waste = Not Served + Plate Waste
- Share Eaten: the share of the food on the pupil’s plates that was eaten by them
  Share Eaten = Eaten/Served
- Share not Served: the share of the wasted food that was not served
  Share not Served = Not Served/Total Wasted.

For each stage, data was recorded separately for the following 4 meal components: soup, protein second dish (meat or fish), starch second dish, and vegetable second dish.

In primary schools, the leftover food on the plates returned by pupils was divided by the project staff into the considered meal components. In the kindergartens, only the ‘food ladies’ had access to the classrooms; they collected the leftovers according to meal components in small containers. The mass of prepared food and food waste at each stage and for each component was determined using calibrated kitchen scales. All measurements were recorded as net weights, excluding container mass.

During the monitoring phase, no active involvement of school or catering staff in data collection was required. However, kitchen personnel facilitated access to production processes and supported the measurement of food quantities where necessary.

## 3. Results

This chapter presents the results of the nudging intervention for primary schools No. 84 and No. 1 in Wrocław and for kindergarten No. 56.

### 3.1. Comparison Baseline Versus Intervention

**Figure 2:**
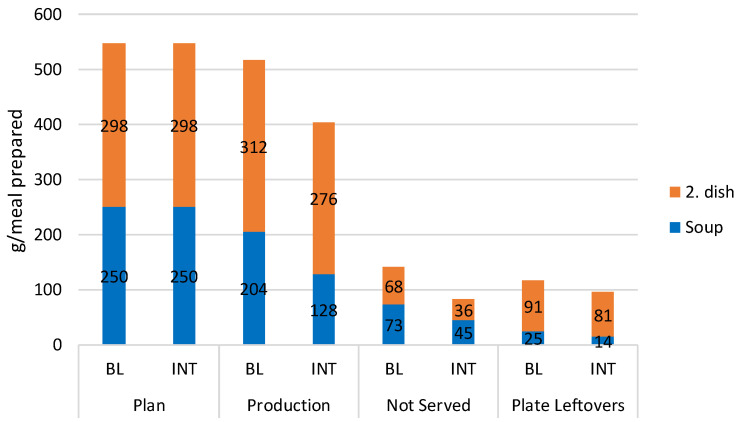
Amounts of soup and second dish produced, not served (incl. serving leftovers), as well as plate waste during the baseline measurement (BL) and the intervention measurement (INT) in primary school no. 84.

**Figure 3:**
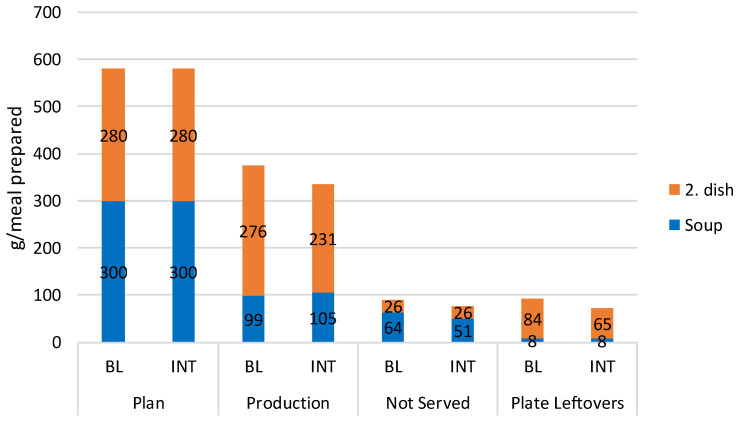
Amounts of soup and second dish produced, not served (incl. serving leftovers), as well as plate waste during the baseline measurement (BL) and the intervention measurement (INT) in primary school no. 1.

**Figure 4:**
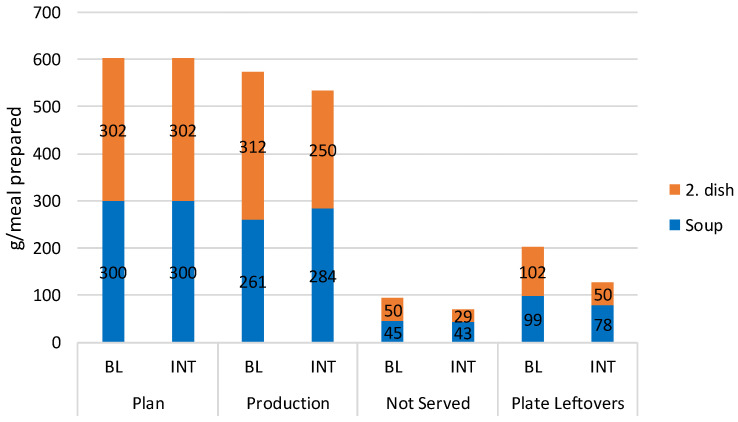
Amounts of soup and second dish produced, not served (incl. serving leftovers), as well as plate waste during the baseline measurement (BL) and the intervention measurement (INT) in kindergarten no. 56.

The first application of the nudging installation occurred in primary school no. 84, followed by kindergarten no. 56 and primary school no. 1, in November and December 2025. The achieved amounts of produced, eaten, and wasted food were compared to the results in the baseline monitoring, which had been carried out exactly one year earlier. Figure 2, Figure 3 and Figure 4 show the resulting amounts for the starter dish (soup, which is always served in Polish schools) and the second dish. In these figures the parameters that were directly measured during both the baseline and intervention phase are shown: amount of food prepared, plate waste, and food prepared but not served (this fraction includes serving leftovers, which are limited).

In Figure 2, the 5-day average amount of food produced and wasted is shown for primary school no. 84. Although the intervention was primarily aimed at the second dish, pupils were very keen on participating with their soup plates as well. For this reason, the results for the soup are also shown in this study. It can be clearly seen that the amounts of soup and second dish produced, which should have been identical for both the baseline and intervention measurement, were strongly reduced. During the baseline measurement, the second dish amount was slightly above plan and during the intervention it was below. For the soup, the difference was greater. Between the baseline measurement (November 2024) and the intervention measurement (November 2025) a new catering company was engaged by the school direction. The new company was very committed to eliminating food waste and tried to minimize the amount of not served food in the school canteen. At the request of the study team, the caterer adjusted their menu during the intervention week (to be identical to the baseline week), but the amounts were not quite identical. Therefore, it is not possible to assess the influence of the intervention on the reduction of the unserved food amounts, as lower amounts of produced food automatically go hand in hand with reduced amounts of not served food. For both the soup and the second dish a clear decrease in both the unserved food and the plate waste can be observed: 38% and 45%, respectively, for the soup, while the second dish decreases by 46% and 11%, respectively.

Due to the change of the catering company in primary school no. 84 it was decided to include school no. 1 in the study. In school no. 1 the food is prepared in the school’s kitchen by staff employed by the school. No changes in staff occurred between the measurement periods. Figure 3 shows the effects of the intervention in primary school no. 1.

The amount of prepared soup was significantly lower than the planned quantities both during the intervention period and the baseline. The actual amounts produced are similar during both periods. In the case of the second dish the production during the intervention phase is lower than the planned and lower than during the baseline. In general, in primary schools, soup waste is dominated by the unserved portions, whereas the second dish is mostly wasted as plate waste. The reason for this is that especially the older pupils (not accompanied by a supervisor) take soup voluntarily and consequently often skip it. As a result, the soup does not leave the kitchen and is reported as not served.

For the second dish, the amount of food not served stayed largely unchanged, while the amount of plate waste showed a decrease of 22%. In the case of soup, the plate waste remained unchanged, whereas the amount of unserved food decreased by 20%. Due to the reduced production, it is not possible to say clearly what caused the reduction of unserved food.

In the foodCIRCUS project, kindergartens were monitored in addition to primary schools. In Figure 4 the effects of the intervention in kindergarten no. 56 are shown for the seven classes in which the intervention was applied.

In kindergarten no. 56 the difference between planned and actually prepared food was smaller than in the primary schools. The production of soup increased slightly during the intervention period, whereas the amount of the second dish prepared was significantly lower.

The unserved soup changed only slightly during the intervention period, while the plate waste was reduced by 21%. For the second dish a clear decrease in wasted amounts could be observed: a 43% reduction in not served amounts and a 51% reduction for the plate waste.

In addition to the amounts measured directly (production, food not served, and plate waste), the average amount of food served, the average amount of food eaten, and the total amount of food wasted were also determined. Additionally, two target parameters were defined to determine the success of the intervention:

- Share Eaten—the proportion of food on the pupil’s plates that was eaten by them (Eaten/Served)
- Share not Served—the proportion of the wasted food that was not served (Not Served/Total Wasted).

In Table 2 all these parameters for both the baseline (BL) and intervention (INT) period as well as the difference (DIFF) between them are provided. For the parameters Share Eaten and Share not Served, the difference is presented as percentage points. As soup was not the primary target of the intervention, only the results for the second dish are shown, divided into protein, starch, and vegetables fractions.

It could be shown that the amount of second dish produced was significantly reduced during the intervention period. However, looking at the detailed components of the second dish, it can be seen that the main reduction in primary school no. 84 is in the vegetables, in school no. 1 in the starch fraction, and in kindergarten no. 56 in the protein fraction (Table 2).

In primary school no. 84, the reduced amount of food prepared was followed by a strong reduction of food not served (32 g/meal or 46%), whereas the portions served were only slightly smaller (5 g/meal or 2%). Of these served portions, slightly more was eaten (5 g/meal or 3%), leading to a reduction of the plate waste of 10 g per meal (11%).

On the other hand, in primary school no. 1, the reduction in food produced did not lead to a reduction in food not served. The portions served were significantly smaller during intervention (45 g/meal or 18%). The amount of food eaten was also reduced by 27 g/meal (16%). This resulted in remaining plate waste which was 19 g less per meal (22%) after the nudging.

For the seven observed classes of kindergarten no. 56, the reduction in produced food was the largest amongst the participants (62 g/meal). At the same time the pupils ate more (12 g/meal or 7%). As the served portions were reduced by 40 g per meal (15%), it led to a strong decrease of both the food not served (22 g/meal or 43%) and the plate waste (52 g/meal or 51%).

The meal fractions with the largest relative change between the baseline and intervention period were the vegetables not served in primary school no. 84 (reduction of 23 g/meal or 74%) and the starch component left on the pupil’s plates in kindergarten no. 56 (reduction of 34 g/meal or 66%).

The main objectives of the nudging intervention—an increase of the proportion of served food to be eaten (Share Eaten) and an increase of the share of not served food in the total wasted food (Share not Served)—were not completely met. The proportion of the served food being eaten increased slightly in both primary schools (3 respectively 2 percentage points) and more significantly in the kindergarten (16 percentage points). The Share not Served was increased in school no. 84 (by 12 percentage points) but was reduced in school no. 1 and in the kindergarten (by 5 and 3 percentage points respectively). The latter indicator, especially, is not only influenced by how much the pupils put on their plates but also by the amount of food produced. As the amount of food produced was significantly reduced during the intervention period, it cannot be unequivocally concluded that the nudging installation leads to a shift from plate waste to food not served (which is desirable, as the latter can be used for redistribution purposes).

### 3.2. Comparison Intervention Group Versus Control Group in Kindergarten No. 56

Kindergartens in Poland accept children roughly at the age of 3–6 years. Kindergarten no. 56 consists of three buildings at two separate locations, with one kitchen used at each location. For organizational reasons, it was only possible to monitor food waste generation in one location (kitchen), consisting of two buildings. This implies that in order to determine the effects of the intervention, the nudging was installed only in half of the classes (the classes which had pupils that were present already in November 2024). During both the baseline and intervention period, the monitoring was also carried out in seven classes in which no intervention was planned. The results thereof can however only be considered as a quasi-control group, as the pupils that were present during the baseline period had already left kindergarten during the intervention period. The seven classes (not part of the intervention itself) at that time consisted of new pupils.

In Table 3 the results of the nudging intervention in kindergarten no. 56, comparing the intervention and quasi control classes, are summarized.

The results in Table 3 show that the total amount of food produced is significantly lower when comparing the intervention to the baseline period for both groups. At the same time the total amount of food waste generated decreased more than the decrease in production for the intervention group. For the quasi-control group, the total waste decreased less than the decrease in production. The pupils in the intervention group ate more (12 g/meal or 7%), whereas in the control group, the amount of food eaten decreased (38 g/meal or 22%). With the decreasing amounts of food served for both groups, this results in an increase of the Share Eaten (calculated as the amount Eaten divided by the amount Served) for the intervention group of 16 percentage points. For the control group, there is a minor decrease to be seen. The indicator Share not Served (the part of the wasted food that is not served) slightly increased in the control group, whereas in the intervention group, it slightly decreased. The amount of plate waste was higher in the intervention group compared to the control group during the baseline measurement before the intervention. After the intervention the situation was the other way around. The amount of plate waste decreased in both groups: in the intervention group by 52 g per meal (51%), in the control group by 14 g per meal (20%).

## 4. Discussion

For all educational institutions considered, the amount of food prepared was lower than the planned amounts. This is especially true for the soup in both primary schools. The reason for this large difference is the fact that soup is offered to all pupils who eat lunch, but only a few partake. Since it is optional, many pupils do not take soup and immediately start with the second dish. The limited time that the pupils have to eat their lunch is a factor that does not support the eating of soup.

In both primary schools and in the kindergarten the overall waste level (soup and second dish combined) during the baseline compared to the literature was high [12,15,37,38,39,40], with a level of 257 g/meal prepared for primary school no. 84, 183 g/meal for school no. 1, and 297 g/meal for kindergarten no. 56. In all cases, this level decreased to 177, 150, and 200 g/meal prepared during the intervention period. The equates to a 31% reduction in food waste for primary school no. 84, 18% for school no. 1, and 33% for kindergarten no. 56. These effects are also high, compared to the literature values for nudging intervention strategies [37,39,40,41,42,43]. However, as the amounts of prepared food were not identical during the baseline and intervention periods, these reduction effects can only partly be ascribed to the effect of the nudging. None of the nudging interventions in the literature use a ball-voting installation, however.

Focusing on the main target for the nudging intervention, it can be concluded for the second dish that the production was lower in all schools during the intervention period, although these amounts were intended to be identical during the two measurement periods. For primary school no. 84 and kindergarten no. 56, the reduction in total food waste was higher than the reduction in the produced amount of food. This is an indication that the nudging installation is effective. In school no. 1, however, the effect was opposite.

The nudging installation encourages the pupils to leave less food on their plates. To achieve this, they can either eat more or take smaller portions. The kitchen staff was instructed to follow the children’s wishes on the portions served. Plate waste decreased in all considered educational institutions: by 10 g/meal (11%) in primary school no. 84, by 19 g/meal (22%) in primary school no. 1, and by 52 g/meal (51%) in kindergarten no. 56. At the same time the amount of food actually eaten by the pupils in the two schools increased slightly and decreased significantly in one school. The resulting indicator, the share of the average portion of food served that was actually eaten, increased slightly for primary schools no. 84 and no. 1 (3 and 2 percentage points, respectively) and significantly for primary school no. 56 (16 percentage points). Therefore, the nudging installation tends to steer pupils in the direction of ‘putting on their plate what they will eat’.

Another objective of the intervention was to create a shift from plate waste to not served waste, as the latter part of the overproduction can be avoided by preparing less, or used for redistribution, whereas the plate waste cannot. The indicator Share not Served (food not served divided by the total amount of food wasted) increased clearly for school no. 1 but decreased slightly for the other schools. Based on these results, no clear conclusions can be drawn. As the amount of food prepared decreased, it had a direct impact on the amount of resulting food not served. To properly determine the effects on the Share not Served, the production level should remain constant.

For individual meal components, the picture was varied, with different components showing different effects in the educational institutions where the research was conducted.

Another way to assess the effects of food waste interventions in schools is the consideration of control groups, in which no intervention is implemented. In this study, half of the kindergarten pupils were in the intervention group, the other half in a quasi-control group. This is because the pupils in the latter group were new. The ones present during the baseline had left kindergarten and their place had been taken by new pupils. In the intervention group classes, the plate waste was reduced significantly more than in the control group classes. In the intervention group, the share of the food served that was actually eaten also significantly increased, whereas in the control group this effect was not observed. This is another indication of the effectiveness of the nudging intervention.

## 5. Conclusions

The present study enables the following conclusions to be made:

- A novel nudging intervention method was applied in three education institutions
- All institutions initially had a high level of food wastage (*soup and second dish* combined)—257 g/meal prepared for primary school no. 84, 183 g/meal for school no. 1, and 297 g/meal for kindergarten no. 56
- In all institutions this level decreased significantly during the nudging intervention—by 31% for primary school no. 84, 18% for school no. 1, and 33% for kindergarten no. 56. This reduction, however, is caused by both a reduction in prepared amounts and by the nudging
- The amount of second dish food actually eaten by the pupils in the two schools slightly increased and in one school significantly decreased
- The second dish plate waste decreased in all the considered schools—by 10 g/meal (11%), 19 g/meal (22%), and 52 g/meal (51%), respectively,
- After the intervention a larger share of the served second dish was eaten than was left on the plates, in comparison to the situation during the baseline monitoring
- A shift from plate waste to not served food, which was one of the study aims, could not be unambiguously confirmed
- The nudging intervention was effective, which was also confirmed by comparison to the quasi-control group
- Significant changes in food production amounts hamper the possibility to determine the effects of the nudging intervention.

Based on the experiences in the current study, the following can be recommended for further nudging intervention studies:

- Keep the food production level at exactly the same level during the baseline and intervention periods
- Determine the long term effects of the nudging intervention (planned for 2026)
- Give pupils maximal freedom to decide about portion sizes during the nudging intervention
- Carry out baseline and intervention monitoring studies within the same school year. This avoids changes in caterers and kitchen staff, and it enables the intervention and control groups to have the same pupils for the whole period of the study.

## Figures and Tables

**Table 1:** Number of prepared meals and monitoring plan in the participating education institutions.

Institution	Meals Prepared Baseline [av. no./d]	Meals Prepared Intervention [av. no./d]	Monitoring Baseline	Monitoring Intervention
PS84	353	380	4–8 November 2024	3–7 November 2025
PS1	455	471	2–6 December 2024	1–5 December 2025
KG56, intervention	135	124	25–29 November 2024	24–28 November 2025
KG56, no intervention	148	153	25–29 November 2024	24–28 November 2025

**Table 2:** Effects (g/meal prepared) of the nudging intervention in the three considered Wrocław schools.

		Primary School No. 84				Primary School No. 1				Kindergarten No. 56			
		protein	starch	vegetables	**total**	protein	starch	vegetables	**total**	protein	starch	vegetables	**total**
Production	BL	109	135	68	**312**	45	163	68	**276**	86	150	77	**312**
	INT	110	137	30	**276**	42	129	60	**231**	56	128	66	**250**
	DIFF	1	2	−39	**−36**	−3	−33	−9	**−45**	−30	−22	−10	**−62**
Not Served	BL	19	18	31	**68**	3	17	6	**26**	12	25	14	**50**
	INT	14	14	8	**36**	2	17	7	**26**	3	14	11	**29**
	DIFF	−5	−4	−23	**−32**	−1	0	1	**0**	−8	−10	−3	**−22**
Plate Waste	BL	36	38	17	**91**	10	50	24	**84**	20	52	31	**102**
	INT	37	35	9	**81**	8	39	18	**65**	14	18	18	**50**
	DIFF	1	−2	−8	**−10**	−2	−10	−6	**−19**	−5	−34	−13	**−52**
Served	BL	90	117	37	**244**	41	146	62	**250**	74	125	63	**262**
	INT	95	123	22	**240**	40	112	53	**204**	53	113	56	**222**
	DIFF	5	6	−16	**−5**	−2	−34	−10	**−45**	−21	−12	−7	**−40**
Eaten	BL	54	79	20	**153**	31	96	39	**166**	55	73	31	**160**
	INT	58	88	13	**159**	32	73	35	**139**	39	96	37	**171**
	DIFF	5	8	−7	**5**	0	−23	−4	**−27**	−16	22	6	**12**
Total Wasted	BL	55	56	48	**159**	13	67	30	**110**	31	76	45	**153**
	INT	51	49	17	**117**	10	57	25	**92**	18	32	29	**79**
	DIFF	−4	−6	−31	**−41**	−4	−10	−5	**−18**	−14	−44	−16	**−74**
Share Eaten	BL	60%	68%	54%	**63%**	75%	66%	62%	**66%**	74%	59%	50%	**61%**
	INT	61%	71%	60%	**66%**	80%	65%	66%	**68%**	73%	84%	67%	**77%**
	DIFF	1%	3%	5%	**3%**	5%	−1%	4%	**2%**	−1%	26%	17%	**16%**
Share not Served	BL	65%	68%	36%	**57%**	76%	75%	80%	**76%**	62%	68%	70%	**67%**
	INT	72%	72%	51%	**69%**	80%	70%	72%	**71%**	81%	55%	63%	**64%**
	DIFF	7%	4%	16%	**12%**	4%	−5%	−8%	**−5%**	19%	−13%	−6%	**−3%**

**Table 3:** Effects of the nudging intervention (g/meal prepared) in 7 intervention classes and 7 quasi control classes of kindergarten no. 56.

		Intervention				Quasi Control			
		protein	starch	vegetables	**total**	protein	starch	vegetables	**total**
Production	BL	86	150	77	**312**	82	129	76	**287**
	INT	56	128	66	**250**	57	107	60	**224**
	DIFF	−30	−22	−10	**−62**	−25	−23	−16	**−64**
Not Served	BL	12	25	14	**50**	9	11	22	**42**
	INT	3	14	11	**29**	9	14	8	**30**
	DIFF	−8	−10	−3	**−22**	−1	3	−14	**−11**
Plate Waste	BL	20	52	31	**102**	19	33	18	**71**
	INT	14	18	18	**50**	13	27	17	**57**
	DIFF	−5	−34	−13	**−52**	−6	−6	−2	**−14**
Served	BL	74	125	63	**262**	73	118	54	**246**
	INT	53	113	56	**222**	49	93	52	**194**
	DIFF	−21	−12	−7	**−40**	−24	−26	−2	**−52**
Eaten	BL	55	73	31	**160**	54	86	36	**175**
	INT	39	96	37	**171**	35	66	36	**137**
	DIFF	−16	22	6	**12**	−18	−19	0	**−38**
Total Wasted	BL	31	76	45	**153**	29	44	40	**112**
	INT	18	32	29	**79**	22	40	24	**87**
	DIFF	−14	−44	−16	**−74**	−7	−3	−15	**−25**
Share Eaten	BL	74%	59%	50%	**61%**	73%	72%	66%	**71%**
	INT	73%	84%	67%	**77%**	73%	71%	68%	**71%**
	DIFF	−1%	26%	17%	**16%**	−1%	−1%	2%	**−1%**
Share not Served	BL	62%	68%	70%	**67%**	68%	75%	46%	**63%**
	INT	81%	55%	63%	**64%**	61%	66%	68%	**65%**
	DIFF	19%	−13%	−6%	**−3%**	−7%	−9%	22%	**2%**

## Data Availability

The original contributions presented in this study are included in the article. Further inquiries can be directed to the corresponding author.

## References

[1] Kohli K., Prajapati R., Shah R., Das M., Sharma B.K. (2024). Food Waste: Environmental Impact and Possible Solutions. Sustain. Food Technol..

[2] Stoica D., Zlati M.L., Bălan (Stanciu) R., Bălănică-Dragomir C.M., Bichescu C.I., Dragomir-Constantin F.-L., Stoica M. (2025). Plate Food Waste in Early Childhood Education: Contextual and Nutritional Drivers with Implications for Sustainable Food Systems. Foods.

[3] World Bank (2020). Addressing Food Loss and Waste: A Global Problem with Local Solutions.

[4] Food and Agriculture Organization of the United Nations (2013). Food Wastage Footprint: Impacts on Natural Resources—Summary Report.

[5] Rodríguez Palleres X., Villota Arcos C., Toledo San Martín Á., Rojas González F., Castagnini J.M. (2024). Fruit and Vegetables Loss and Waste in Preschools Belonging to the National Board of Kindergartens of Chile. An. Sist. Sanit. Navar..

[6] Eurostat Food Waste and Food Waste Prevention—Estimates—Statistics Explained—Eurostat. https://ec.europa.eu/eurostat/statistics-explained/index.php?title=Food_waste_and_food_waste_prevention_-_estimates.

[7] European Commission (2023). SWD(2023) 421 Final—Part 3/4—COMMISSION STAFF WORKING DOCUMENT IMPACT ASSESSMENT REPORT Accompanying the Document Directive of the European Parliament and of the Council Amending Directive 2008/98/EC on Waste.

[8] Cáceres Sandoval M.L., Cote Daza S.P. (2025). Development of a Strategy to Reduce Food Waste in a Preschool Food Service. Sustainability.

[9] United Nations Environment Programme (2024). Food Waste Index Report 2024.

[10] Wani N.R., Rather R.A., Farooq A., Padder S.A., Baba T.R., Sharma S., Mubarak N.M., Khan A.H., Singh P., Ara S. (2023). New Insights in Food Security and Environmental Sustainability through Waste Food Management. Environ. Sci. Pollut. Res..

[11] Derqui B., Grimaldi D., Fernandez V. (2020). Building and Managing Sustainable Schools: The Case of Food Waste. J. Clean. Prod..

[12] Byker C.J., Farris A.R., Marcenelle M., Davis G.C., Serrano E.L. (2014). Food Waste in a School Nutrition Program After Implementation of New Lunch Program Guidelines. J. Nutr. Educ. Behav..

[13] Boschini M., Falasconi L., Giordano C., Alboni F. (2018). Food Waste in School Canteens: A Reference Methodology for Large-Scale Studies. J. Clean. Prod..

[14] Cordingley F., Stephenson J., Reeve S. (2011). Food Waste in Schools.

[15] Falasconi L., Boschini M., Giordano C., Cicatiello C., Alboni F., Nassivera F., Troiano S., Marangon F., Segrè A., Franco S. (2025). Who Cleans the Plate? Quantity and Type of Food Waste in 78 Primary Schools’ Canteens in Italy. Sustainability.

[16] Filimonau V., Ermolaev V.A., Vasyukova A. (2022). Food Waste in Foodservice Provided in Educational Settings: An Exploratory Study of Institutions of Early Childhood Education. Int. J. Gastron. Food Sci..

[17] Capper T., Brennan S., Woodside J., McKinley M. (2022). What Makes Interventions Aimed at Improving Dietary Behaviours Successful in the Secondary School Environment? A Systematic Review of Systematic Reviews. Public Health Nutr..

[18] Li J., Li W., Wang L., Jin B. (2021). Environmental and Cost Impacts of Food Waste in University Canteen from a Life Cycle Perspective. Energies.

[19] Filimonau V., Lemmer C., Marshall D., Bejjani G. (2017). ‘Nudging’ as an Architect of More Responsible Consumer Choice in Food Service Provision: The Role of Restaurant Menu Design. J. Clean. Prod..

[20] Guthrie J., Mancino L., Lin C.J. (2015). Nudging Consumers toward Better Food Choices: Policy Approaches to Changing Food Consumption Behaviors. Psychol. Mark..

[21] Gardner G., Burton W., Sinclair M., Bryant M. (2023). Interventions to Strengthen Environmental Sustainability of School Food Systems: Narrative Scoping Review. Int. J. Environ. Res. Public Health.

[22] Thaler R.H., Sunstein C.R. (2009). Nudge: Improving Decisions About Health, Wealth and Happiness.

[23] Kuyer P., Gordijn B. (2023). Nudge in Perspective: A Systematic Literature Review on the Ethical Issues with Nudging. Ration. Soc..

[24] Amiri B., Jafarian A., Abdi Z. (2024). Nudging towards Sustainability: A Comprehensive Review of Behavioral Approaches to Eco-Friendly Choice. Discov. Sustain..

[25] Viale R. (2022). Nudging.

[26] Bucher T., Collins C., Rollo M.E., McCaffrey T.A., De Vlieger N., Van der Bend D., Truby H., Perez-Cueto F.J.A. (2016). Nudging Consumers towards Healthier Choices: A Systematic Review of Positional Influences on Food Choice. Br. J. Nutr..

[27] Vecchio R., Cavallo C. (2019). Increasing Healthy Food Choices through Nudges: A Systematic Review. Food Qual. Prefer..

[28] Wilson A.L., Buckley E., Buckley J.D., Bogomolova S. (2016). Nudging Healthier Food and Beverage Choices through Salience and Priming. Evidence from a Systematic Review. Food Qual. Prefer..

[29] Lycett K., Miller A., Knox A., Dunn S., Kerr J.A., Sung V., Wake M. (2017). ‘Nudge’ Interventions for Improving Children’s Dietary Behaviors in the Home: A Systematic Review. Obes. Med..

[30] Ferrari L., Cavaliere A., De Marchi E., Banterle A. (2019). Can Nudging Improve the Environmental Impact of Food Supply Chain? A Systematic Review. Trends Food Sci. Technol..

[31] Kroese F.M., Marchiori D.R., de Ridder D.T.D. (2016). Nudging Healthy Food Choices: A Field Experiment at the Train Station. J. Public Health.

[32] Vandenbroele J., Vermeir I., Geuens M., Slabbinck H., Van Kerckhove A. (2020). Nudging to Get Our Food Choices on a Sustainable Track. Proc. Nutr. Soc..

[33] von Kameke C., Fischer D. (2018). Preventing Household Food Waste via Nudging: An Exploration of Consumer Perceptions. J. Clean. Prod..

[34] Papargyropoulou E., Wright N., Lozano R., Steinberger J., Padfield R., Ujang Z. (2016). Conceptual Framework for the Study of Food Waste Generation and Prevention in the Hospitality Sector. Waste Manag..

[35] Kallbekken S., Sælen H. (2013). ‘Nudging’ Hotel Guests to Reduce Food Waste as a Win–Win Environmental Measure. Econ. Lett..

[36] Wilkie A., Graunke R., Cornejo C. (2015). Food Waste Auditing at Three Florida Schools. Sustainability.

[37] Lonska J., Kodors S., Deksne J., Litavniece L., Zvaigzne A., Silicka I., Kotane I. (2025). Reducing Plate Waste in Latvian Schools: Evaluating Interventions to Promote Sustainable Food Consumption Practices. Foods.

[38] Tóth A.J., Kunszabó A., Szakos D., Battay M.B., Süth M., Kasza G., Bittsánszky A. (2026). Challenges in Promoting Pro-Environmental Behaviour to Reduce Food Waste in Schools. Waste Manag..

[39] Persson Osowski C., Osowski D., Johansson K., Sundin N., Malefors C., Eriksson M. (2022). From Old Habits to New Routines—A Case Study of Food Waste Generation and Reduction in Four Swedish Schools. Resources.

[40] Sundin N., Malefors C., Strotmann C., Orth D., Kaltenbrunner K., Obersteiner G., Scherhaufer S., Sjölund A., Persson Osowski C., Strid I. (2024). Sustainability Assessment of Educational Approaches as Food Waste Prevention Measures in School Catering. J. Clean. Prod..

[41] Malefors C., Sundin N., Tromp M., Eriksson M. (2022). Testing Interventions to Reduce Food Waste in School Catering. Resour. Conserv. Recycl..

[42] Vidal-Mones B., Diaz-Ruiz R., Gil J.M. (2022). From Evaluation to Action: Testing Nudging Strategies to Prevent Food Waste in School Canteens. Waste Manag..

[43] Jaworski M., Chojnowska E. (2026). The ‘Schools Don’t Waste’ Program: A Theory-Informed Participatory Intervention to Reduce Plate Waste in Public School Canteens. Nutrients.

